# The search for the best infantry boot

**DOI:** 10.1186/s40696-016-0024-5

**Published:** 2016-10-10

**Authors:** Charles Milgrom, Alex Sorkin, Arnon Gam, Jonathan Singer, Itamar Nir, Boris Kogan, Aharon S. Finestone

**Affiliations:** 1 Hebrew University Medical School, 91120 Ein Kerem, Jerusalem, Israel; 2 IDF Medical Corps, Jerusalem, Israel; 3 Orthopaedic Surgery Service, IDF Medical Corps, Jerusalem, Israel; 4 Tzameret Military Medical Track, Hebrew University Medical School, Jerusalem, 91120 Israel; 5 Technological and Logistics Branch, IDF, Jerusalem, Israel; 6 Assaf HaRofeh Medical Center, Affiliated to The Sackler Faculty of Medicine, Tel Aviv University, 70300 Zerifin, Israel; 7 IDF Medical Corps, Active Reserve, Jerusalem, Israel

**Keywords:** Infantry boots, Recruits, Boot longevity, Foot injuries, Boot comfort

## Abstract

**Background:**

The combat role of the twenty-first century infantry soldier has changed and accordingly their boots should evolve to meet these new needs and maximize soldier performance.

**Objective:**

To evaluate injuries and durability of the hot weather infantry boots (HWIB) in elite infantry training and assess the initial performance of newly designed Israeli infantry boots (NDIB).

**Methods:**

In Phase 1, the durability of the HWIB during elite infantry training was evaluated at weeks 10, 19 and 64 in a cohort of 67 recruits. At each exam recruits removed their boots which were assessed for wear and integrity and photographed. The number of times recruits changed their boots was recorded. In Phase 2, foot injuries were assessed in a cohort of 73 elite infantry recruits wearing HWIB. In Phase 3, 65 infantry recruits were issued the NDIB. Recruits feet were measured for width and shoe size using the Brannock device and then followed for problems associated with their boots. Foot lesions were document by photographs.

**Results:**

Phase 1: The mean longevity of HWIB in training was 5.2 ± 0.2 (SE) months, (95 % CI 4.83–5.61). Phase 2: 38 % of the elite infantry recruits wearing HWIB had at least one complaint and 31 (42 %) were found to have boot related injuries in a total of 56 injured areas. Phase 3: The mean predicted boot size (42.8 ± 1.7) based on Brannock measurements, was less than the size of the NDIB actually worn, 43.1 ± 1.6. Only 34.8 % of the feet were width D (the standard shoe width). At 9 day follow up, 55 of the 65 recruits who wore NDIB reported at least one problem with them (85 %, p < 0.0001, compared to HWIB). By 3 weeks, all but five recruits had returned to wearing the HWIB. Of the recruits wearing NDIB, 47 (72 %) were found to have had at least one boot related injury with a total number of 180 injured foot areas (p = 0.0004, compared to HWIB).

**Conclusions:**

The HWIB was well tolerated by the elite infantry recruits and associated with significantly less foot injuries than the NDIB. The longevity of the HWIB in demanding elite infantry training was five months.

*Trial registration*: NCT02810002 retrospectively registered June 22, 2016

## Background

The operational tasks and logistics of modern infantry soldiers are constantly changing to accommodate to new combat needs. Infantry soldiers are becoming increasingly specialized. They carry and operate more sophisticated weaponry, carry intelligence and communication systems and wear body armor [1]. Some of their traditional activities has been augmented or changed by technology. Infantry boot design and materials should answer to these new needs so as to maximize soldier performance. They need to be sufficiently comfortable so as not to interfere with training and combat activity.

To design infantry boots optimal for all infantry soldiers in all of their training and combat conditions is a challenge. General factors that need to be considered in boot design include: comfort, weight, water resistance, breathability, traction, durability, foot anatomy, foot protection, operational climate and cost. A partial solution to these variables in the United States Army has been achieved by issuing separate 2 lb. (0.9 kg) hot weather and 2.5 lb. (1.1 kg) temperate weather boots to their soldiers.

Several years ago the Israel Defense Forces (IDF) Technological and Logistics Branch (TLB) changed the standard army boots in an attempt to answer the changing needs of their infantry soldiers. The bilayer rubber sole boots manufactured on lasts designed by the IDF in the 1990s [2] was replaced with the hot weather infantry boots (HWIB) used by the US Army and, manufactured in the USA. The US HWIB are not traditional army boots and have features that are borrowed from athletic shoes. The uppers are made of both soft leather and nylon. The sole is bilayer, with an inner layer of polyurethane and an outer covering of rubber for good shock absorption and traction with low weight [3, 4]. The boot has removable inserts. These features make the boot lighter and more comfortable than traditionally designed military boots and more akin to a high top athletic shoe in its properties [5].

Based on their experience with IDF soldiers, an Israeli manufacturer designed new boots on a new last using bilayer rubber soles (NDIB). They intended their boot to serve the needs of the IDF soldiers better than the HWIB both in durability and comfort.

After several years of successful use of the HWIB, the IDF decided to evaluate the durability of the boots in a field study (Phase 1) and in Phase 2 assess the foot injuries associated with HWIB use. Phase 3 was designed to compare the HWIB with the NDIB for comfort, injuries and durability. We report the results of these three studies.

## Methods

### Phase 1: Durability study of the HWIB

This study was an observational, long term prospective study of the durability of the HWIB supplied to the IDF by the Belleville Boot Company (Belleville IL, USA). Its design was based on their 390 Trop St, with adaptations according to IDF TLB-specifications. Elite infantry recruits (n = 67) who began their basic training in 2013 were enrolled in the study. Their mean age was 19.5 ± 0.9, weight 75.2 ± 7.2 kg, and boot size 43.6 ± 1.5. Prior to beginning their basic training subjects had a general orthopaedic exam to determine that they had no orthopaedic abnormalities that might cause abnormal wear of their boots. All data were recorded in a laptop computer using a custom designed Access^®^ (Microsoft, Redmond WA) application. Each recruit was issued two pairs of HWIB before beginning basic training. Recruits were examined at the first week of basic training and at weeks 10, 19 and 64 of training. At each exam recruits removed their boots to allow inspection of their boots. The boots were assessed for boot sole and upper wear and integrity. Boots were photographed to document their condition. The physical characteristics of the HWIB worn in Phase 1 to 3 of the study and the NDIB used in Phase 3 of the study are shown in Table 1. At each review recruits were questioned whether they were still using their originally issued boots, and if not how many times they had changed boots. A recruit whose boots were worn out or needed a size change presented his boots to the base non-commissioned officer (NCO) in charge of boot logistics. If the NCO agreed that the boots required changing, the damaged boots were sent to a central supply unit for exchange. The recruit then began to use his reserve pair of boots. Receiving new boots takes approximately 4 weeks. Data is presented per boot except where explicitly stated that data refers to pairs of boots.

**Table 1:** Specifications of military boots worn in trials

Specification	HWIB	NDIB
Weight pair boot (gm)	1800 (size 45)	2000 (size 45)
Height (cm, at posterior)	24	22
Inner layer material	Polyurethane	Rubber
Durometer inner layer sole	50-75 Shore A	30-40 Shore A
Outer layer sole material	Rubber Vibram Sierra	Rubber
Durometer outer layer sole	70-80 shore A	50-60 Shore A
Upper leather thickness	2.0 to 2.2 mm single layer-(soft)	2.5 Single layer (hard)
Upper breathable material	1000 Denier nylon	Nylon mesh
Pairs lace closure eyelets	2	4
Pairs lace slide hooks	6 Speed hooks	4 Open hooks
Insole	Removable polyurethane	Removable polyurethane
Waterproof	No	No
Toe cap	Plastic toe cap	Plastic toe cap

### Phase 2: Assessment of foot injuries associated with the HWIB

Assessment of complaints and foot injuries associated with HWIB was performed in a company of 75 elite infantry recruits at weeks 4, 10 and 14 of infantry basic training. All recruits were questioned about foot problems. All recruits removed their shoes and socks and foot lesions were documented. Recruits were also reviewed for overuse injuries.

### Phase 3: Comparison of the HWIB with the NDIB

The HWIB that recruits used in Phase 3 were identical to those described in Phase 1. The NDIB used in Phase 3 were designed and manufactured especially for this study by Brill Shoe Industries Ltd (Rishon LeZion, Israel).

The overall assessment of the NDIB was planned and directed by the TLB and designed to encompass 5 infantry recruit companies inducted in August 2014. All recruits were issued 2 pairs of HWIB at induction before they assembled in their infantry training base. In the study design two recruit companies were scheduled to receive NDIB at week 2 of their basic training and two companies were to remain training in their already issued HWIB. An additional elite infantry company was scheduled for a randomized controlled trial (RCT), recruits being individually randomly assigned to either NDIB or HWIB. The recruits in the elite infantry company were to be followed clinically during their basic training by a team of orthopaedists. IDF institutional review board approval and written informed consent by the recruits in the elite company was required for participation in the study (the other 4 companies were not exposed to any medical procedure and were therefore exempt from IRB approval as in all studies on equipment comfort and longevity performed by the TLB without participation of medical personnel).

Before beginning their training, consenting recruits of the elite company had measurements of weight and height. Foot length and width measurements were made bilaterally using a Brannock measuring device (Brannock Device Company, Liverpool, NY, USA). This device measures the appropriate American shoe size (size 4 to 16) based on foot length. Shoe widths are measured 3A, 2A, A. B, C, D, E, 2E and 3E, progressing from the narrowest to the widest foot. For the same shoe size each increase of shoe width corresponds to a 3/16 inch increase in foot width. The American shoe size numbers were converted to their corresponding European numbers using standard conversion formulas.

Recruits in the elite company were questioned regarding use of the experimental boots allowing for up to 5 complaints. Recruits’ feet were examined and findings recorded directly to the Access^®^ application.

### Statistical analysis

For Phase 1, data of HWIB damage was collected on weeks 10, 19 and 64 and were analyzed per boot and per pair. Data presented in Table 2 relate to the number of boots affected with the type of damage noted for the total number of single boots evaluated in the three visits. The longevity of the boots was calculated based on the total number of pairs of boots that had to be replaced, taking into account the use of the spare pair and also the condition of the present pair of boots (if they were seriously damaged they were counted as replaced). Survival analysis was performed with PROC LIFETEST in SAS using the latest known data, censoring at the time of the latest follow-up if no failure had occurred. Each failure was entered as a separate observation, timed by the number of failures per follow-up time.

**Table 2:** Cumulative HWIB damage observed on inspections at 10, 19 and 64 weeks of training

Affected area	Problem	No. of boots affected
Hind foot area over Achilles tendon	Severely abraded or torn	3 (1.2 %)
Problems involving lace holder (lateral or medial)	Severely abraded or torn	5 (2 %)
Problems with upper including tear not specified above	Severely abraded or torn	37 (15 %)
Inner lining	Torn	1 (0.4 %)
Anterior sole	Split between rubber and PU (delamination)	9 (4 %)
Problems with anterior or posterior part of upper-sole interface	Most of stitches torn or clear separation present between upper and sole	110 Anterior 79 Posterior (45 %, 32 %)
Total areas of damage	Any	244 (100 %)
Total pairs of boots examined		174
Total pairs of boots with damage	Any pair with severe damage in at least one boot	106 (61 %)

Percentage relates to total types of damage (244) and not to total boots with damage, as some boots had more than one type of severe damage

For Phases 2 and 3 up to 5 fields of complaints and/or findings were coded. Complaints not related to the boots (e.g. back pain, knee pain) were not included. Overall complaints (not recruits) are summarized in the tables. The physical examination was encoded on a dedicated Access^®^ sheet with 20 separate areas for each foot. Injuries were classified for each area as none, abrasion, blister, callous, hematoma, redness or wound, with more than one classification possible. Findings in the recruits wearing NDIB that were clearly in the recovery phase (and therefore from before initiation of wearing the NDIB) were disqualified.

Comparison of groups was for total recruits with at least one complaint or at least one finding was estimated with Chi squares (complaints and finding separately).

## Results

### Phase 1

**Fig. 1:**
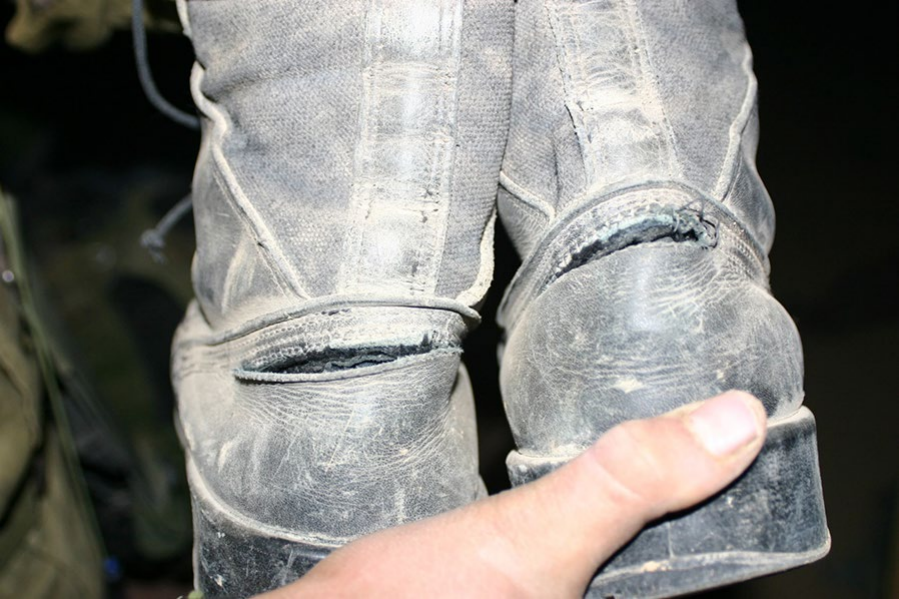
Damage to the heel counter of the HWIB

**Fig. 2:**
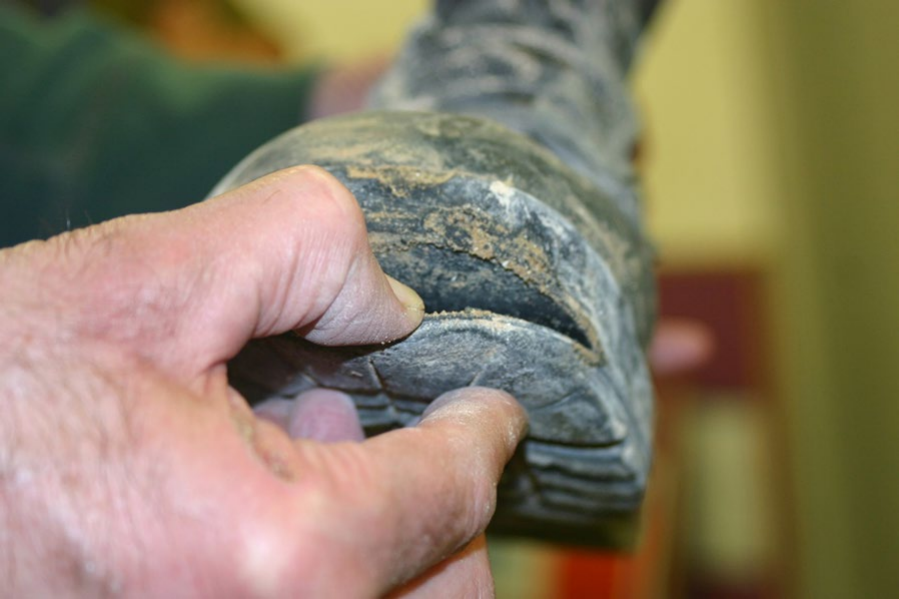
Delamination between the outer rubber and inner polyurethane layers of the HWIB sole

HWIB were examined at 10, 19 and 64 weeks of training. Sixty-four pairs of boots were examined at 10 weeks, 65 pairs at 19 weeks and 45 pairs at 64 weeks of training. The mean longevity of a pair of boots during the training was 5.2 ± 0.2 (SE) months, (95 % CI 4.83–5.61). Table 2 shows the summary of the major boot damage observed at the three observation points during the training. Figure 1 shows an example of a tear in the hind foot region of the boot. Figure 2 shows delamination between the outer rubber and inner polyurethane of the sole.

### Phase 2

Of the 75 elite infantry recruits in the company, 73 were questioned and examined. Twenty-eight (38 %) reported HWIB related problems at the four week follow-up. Twenty-three reported one HWIB related problem and 5 reported two problems. On the basis of physical examination thirty-one (42 %) were found to have at least one boot related injury with a total number of 56 injured areas, 0.77 per recruit.

### Phase 3

All five of the study companies received the HWIB at the time of their induction prior to assembling on their basic training base. According the IRB approved protocol, 98 recruits in the elite infantry company were explained the study details on the Friday of their first week on the base, following which they were given leave home and given an opportunity to consult their family about participating in the study. On their return to base on Sunday they were requested to sign informed consent. Eighteen declined to participate, 79 (81 %) agreed to participate and one was not present because of sick leave. The recruits were available for their anthropometric measurements for only 2 h including a supper break, instead of the scheduled 5 h but measurements of height, weight and foot length and width were completed for all recruits.

The mean age of the recruits who participated in the study from the elite infantry company was 19.4 ± 1.3, their mean height was 174 ± 5.7 cm and their mean weight 70.4 ± 11.1 kg. Table 3 shows the distribution of recruit boot sizes and widths as measured by the Brannock device for these recruits. The mean predicted boot size from the Brannock measurements when converted to European size was size 42.8 ± 1.7. The mean boot size actually worn by recruits was 43.1 ± 1.6. 24.7 % of the feet were width B, 32.5 % of the feet were width C, 34.8 % were width D (the standard shoe width) and 7.9 % width E. Table 3 summarizes the distribution of the measured boot sizes and widths.

**Table 3:** Number of recruits per European shoe size and width converted from measurements using the Brannock device

Boot width	Boot WIDTH						
	39–40	41	42	43	44	45	46
A	0	0	0	0	0	0	1
B	0	0	0	6	6	3	6
C	0	0	2	6	4	12	4
D	0	0	3	12	13	2	1
E	0	2	2	2	0	1	0
2E	1	0	0	0	0	0	0
Total	1	2	7	26	23	18	12

The morning after recruits signed informed consent, boots were distributed. The elite infantry company officers did not agree to a randomized distribution of boots to their recruits. Instead all those recruits in the elite infantry company who agreed to participate were issued NDIB. Additionally, because of a problem of boot sizes there were only enough NDIB to supply one additional non-randomized company. For these logistic reasons the initial study protocol could not be fulfilled and accordingly needed to be modified.

Because of early reports that a number of recruits training with the NDIB were complaining about the boots, the initial scheduled orthopaedic follow up of the elite infantry company was done 9 days after the boot distribution instead of at 3 weeks as originally planned. All recruits filled out a questionnaire relating to the comfort and problems with their boots.

Of the 79 recruits who agreed to participate in the study only 65 actually ever wore the NDIB and were available for follow-up. At the 9 day follow-up twelve recruits who had started to use the NDIB had already discontinued their use. Twenty-eight out of 65 recruits reported one problem with the NDIB. Twenty-two reported 2 problems. Three reported 3 problems and one reported 4 problems associated with boots. The specific recruit complaints of the NDIB group in Phase 3 of the study, compared with those of recruits wearing the HWIB in Phase 2 of the study are presented in Table 4. The number of recruits who reported problems with their shoes was significantly higher among soldiers who wore the NDIB, 55/65 (85 %), than among those who wore the HWIB, 28/73 (38 %), (p < 0.0001), Chi square).

**Table 4:** The recruits’ complaints associated with wearing NDIB in Phase 3 of the study with HWIB in Phase 2 of the study

Problem	Number of complaints (%)	
	HWIB	NDIB
*General complaints*		
Pain	3 (9 %)	3 (3 %)
Not comfortable	0	9 (10 %)
Too stiff	0	3 (3 %)
Too heavy	0	2 (2 %)
No room for orthotics	0	4 (4 %)
Generally tight	3 (9 %)	1 (1 %)
Not suitable for running	1 (3 %)	2 (2 %)
Foot not stable in boot	0	3 (3 %)
Pain on prolonged standing	1 (3 %)	9 (10 %)
Takes time to accommodate	3 (9 %)	0
Too narrow	1 (3 %)	0
*Heel problems*		
Abrasions	11 (33 %)	8 (9 %)
Pressure	4 (12 %)	9 (10 %)
Blisters	0	1 (1 %)
*Midfoot problems*		
Pressure over bunion	1 (3 %)	0
Pressure over 1st MT head	0	11 (12 %)
Pressure over MT heads	2 (6 %)	2 (2 %)
*Toe problems*		
Pressure	1 (3 %)	4 (4 %)
Abrasions	1 (3 %)	3 (3 %)
Blisters	0	1 (1 %)
*Ankle problems*		
Pressure	1 (3 %)	5 (5 %)
Pain	0	1 (1 %)
Sprain ankle	0	1 (1 %)
*Boot lacing system*		
Lacing problems	0	3 (3 %)
Lace hook detached	0	1 (1 %)
Lace associated wounds	0	4 (5 %)
Pressure under hooks	0	1 (1 %)
Pressure at ankle	0	2 (2 %)
*Total number*	33 (100 %)	93 (100 %)
Number of recruits with at least 1 complaint	28 (38 %)	55 (85 %)
Total number of recruits	73	65

**Fig. 3:**
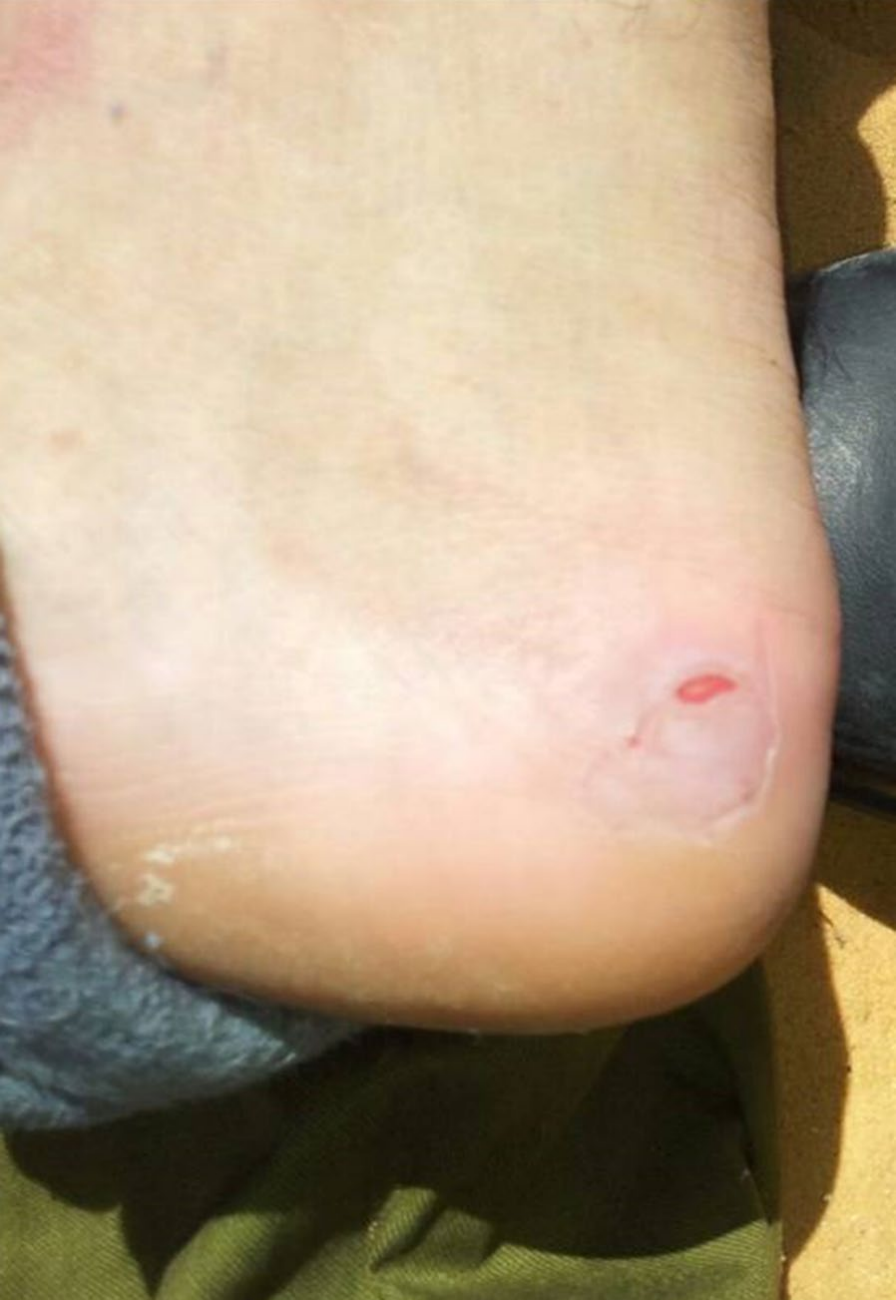
Heel lesion of recruit wearing NDIB

**Fig. 4:**
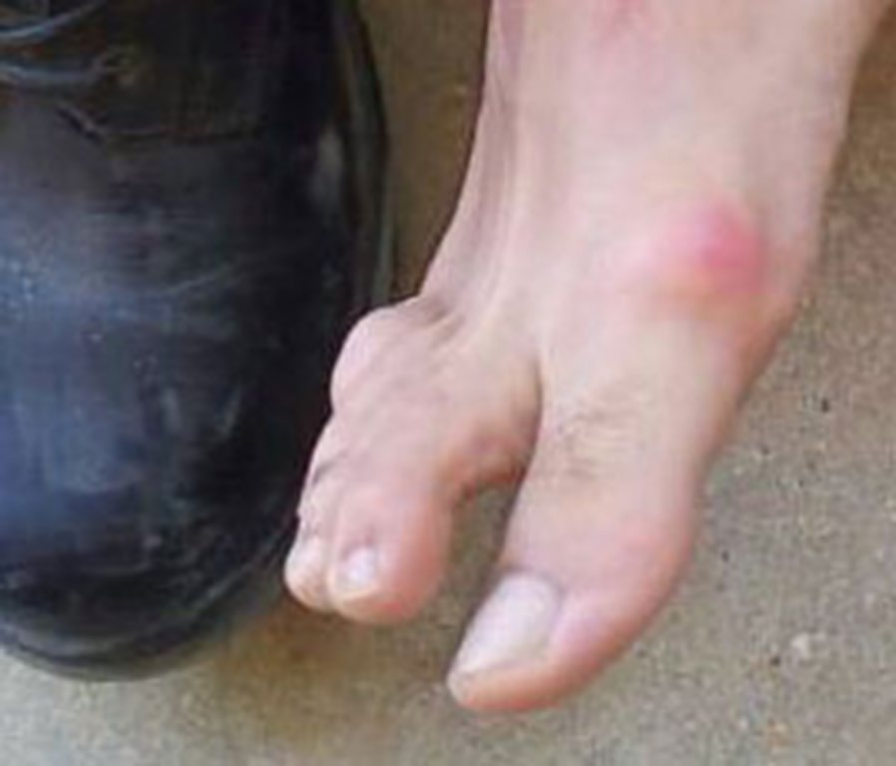
1st metarsophealngeal lesion of recruit wearing NDIB

**Fig. 5:**
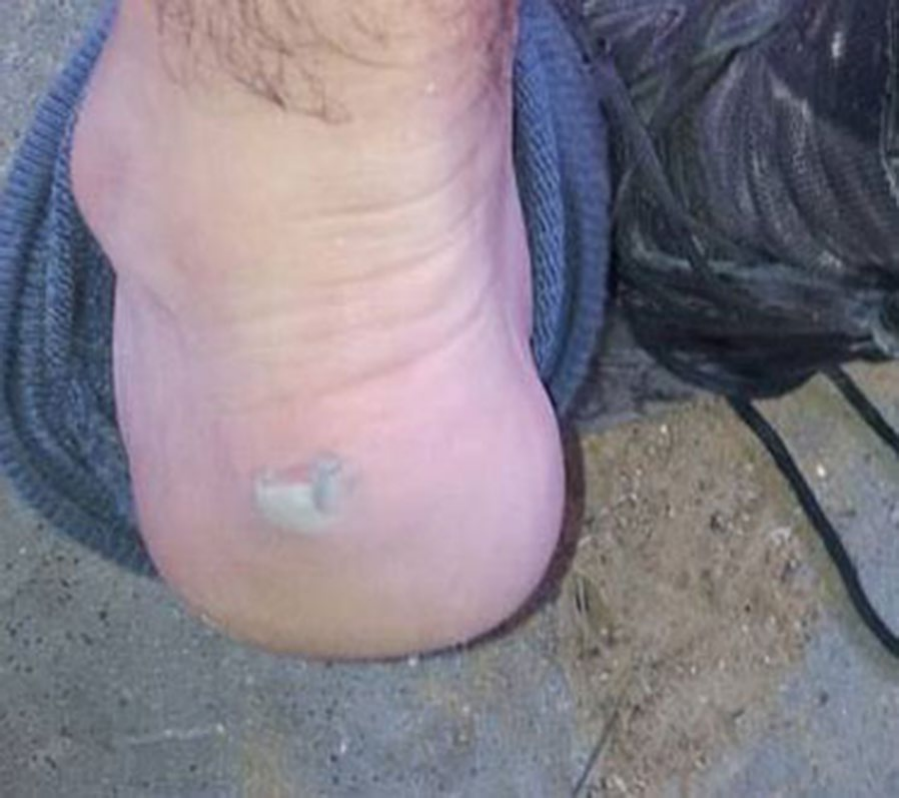
Posterior heel blister of recruit wearing NDIB

The feet of all recruits were examined for skin irritation and wounds. Photographs were taken of all observed skin lesions. Figures 3, 4, 5 show representative lesions found: Fig. 3: a calcaneal lesion (wound); Fig. 4: a 1st metatarsophalangeal joint lesion (redness); Fig. 5: a posterior heel lesion (blister).

**Fig. 6:**
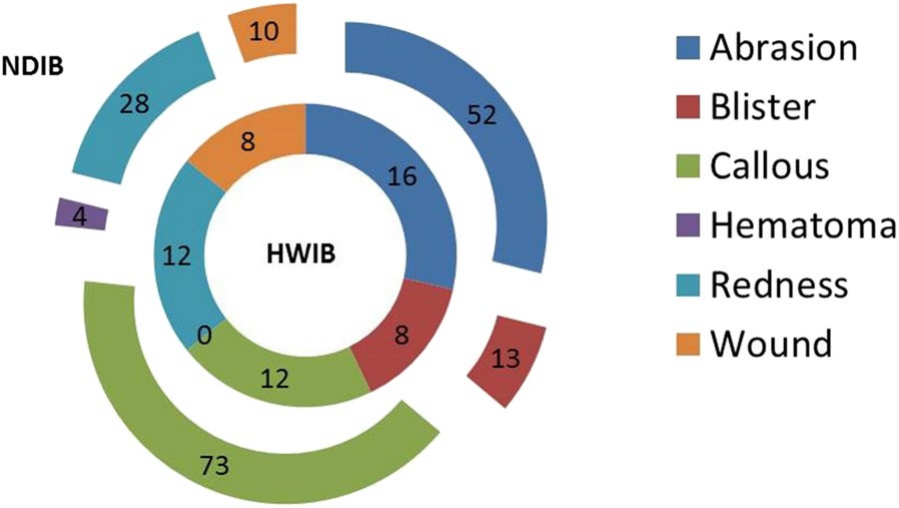
Foot lesion comparison associated with wearing the HWIB (Phase 2) and the NDIB (Phase 3)

**Fig. 7:**
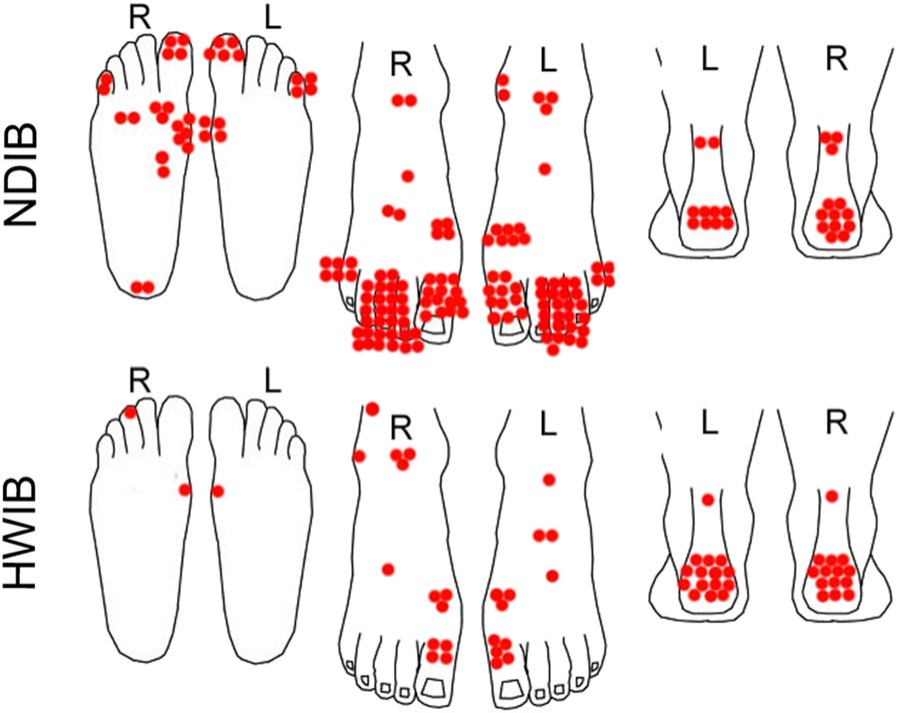
Graphic presentation of the foot lesions in Fig. 6 according to their anatomic location. HWIB indicates the hot weather infantry boot, NDIB indicates the new design infantry boot. Areas classified were (left to right): plantar 5th toe, plantar 2nd to 4th toe, plantar 1st toe, plantar metatarsal heads 2–5, plantar 1st metatarsal head, medial bunion, plantar midfoot, arch, plantar heel, lateral malleolus, anterior ankle, medial malleolus, upper forefoot, upper 2nd to 5th metatarsals, upper 1st metatarsal, upper 5th toe, upper 2nd to 4th toe, upper 1st toe, upper and lower Achilles tendon

Figure 6 shows a comparison of the types of foot lesions identified on physical examination associated with wearing the HWIB in Phase 2 of the study (inner doughnut of the figure) with wearing the NDIB in Phase 3 of the study (outer doughnut of the figure). Forty-seven of the 65 recruits (72 %) wearing NDIB had at least one boot related injury with a total number of 180 injured areas of the foot, compared to 31/73 (42 %) with at least one boot related injury and a total number of 56 injured areas of recruits wearing HWIB, (p = 0.0004, Chi square per recruits with at least one injury). Figure 7 graphically illustrates the anatomical sites of the foot injuries presented in Fig. 6.

Because of the lesions noted in the review and the multitude of complaints, it was decided to allow all recruits to return to training with the HWIB if they wished to do so. Recruits in the second company issued the NDIB were given the option to wear the boot a few days later than the elite infantry company. By this time, the experience of the first company had already filtered through to the second company. Most of the recruits in the second company did not try the boots (a few tried them and quickly returned to the HWIB). At the 5 weeks follow-up only 5 recruits (7.7 %) in the elite infantry company remained training in the NDIB. No complaints of boot problems were reported by any of the control soldiers wearing the HWIB, but their feet were not examined systematically due to the restrictions of our IRB approval and the inability to randomize the volunteers in the company due to the officers’ objection.

## Discussion

In spite of marked methodological flaws, several clear conclusions can be drawn from this study. The HWIB was found to be preferable by elite infantry recruits to the NDIB during their basic training which was in the warm weather. Recruits training with the NDIB had a high number of foot problems associated with their boots from the beginning of their basic training. The HWIB needed to be changed about every five months during elite infantry training because of wear and tear. According to ongoing IDF TLB surveillance of boot returns due to wear and tear indicate HWIB worn by regular infantry soldiers have a boot life of about 10 months.

The HWIB represent a change from the traditional IDF infantry boots which are all seasonal. The HWIB has an upper made from soft leather interspaced with nylon pieces. This makes it breathable and light. The soft upper construction also makes it maximally comfortable when first worn. Traditional IDF boots are made of hard leather which is highly durable and protective but uncomfortable until the boot is “broken in” after some weeks of use. Another factor contributing to the light weight of the HWIB is its sole made from a sandwich of an inner layer of polyurethane for shock absorption and low weight [4] covered by an outer protective rubber layer, much like an athletic shoe. These features make the HWIB more like a high top athletic shoe than a military boot. It is therefore inherently more comfortable and better suited to running and lateral movement than the standard army boot. Each additional 100 g of shoe weight increases wearer physiological energy expenditure during walking by between 7/10ths of a percent to 1 % [6, 7]. Therefore the energy expenditure of a soldier wearing the NDIB is about 2 % higher than if he were wearing the HWIB.

A downside of the hot weather boot is its lower durability and protection against local trauma. The current study did not directly compare the durability of the HWIB with the NDIB. The durability of the HWIB however can be compared with its predecessor in the IDF, the bilayer rubber–rubber sole boot, which has been monitored in a previous study in the same elite infantry basic training by the authors [2], though not yet reported. That boot has the same sole composition and the same thick hard leather as the NDIB. Survival analysis shows that at the end of 14 weeks of training, 67 % of HWIB needed replacement because of shoe damage as compared with 9 % of the bilayer rubber–rubber IDF boot (p < 0.0001, Chi square). Another downside of the HWIB is that it was specifically designed for hot weather use and not for cold, wet weather use. The HWIB performance in the cold weather was not evaluated in the current study and needs to be done.

An infantry soldier in the IDF serves 3 years in compulsory service. After that he is subject to reserve army service for the next 15 or more years. While in compulsory service the soldier has two pairs of boots. After finishing compulsory service an infantry soldier is discharged with a single pair of boots for use in subsequent reserve service. For reserve service, boots need to have a long shelf life and be durable as it is logistically very difficult for the reserve soldier to change boots. This requirement makes the use of HWIB a potential long term problem for IDF reserve soldiers. The sole of the HWIB has an inner layer of polyurethane. This material is known to potentially undergo hydrolysis and disintegrate with time, especially if not stored at a cool temperature. Such storage is likely to be a problem for most reservists in Israel. Indeed even in the short follow up in this study, at week 64, we found that 17 % of the pairs of boots had a split between the rubber and polyurethane layers in the anterior sole in one of the boots.

Because this study monitored the orthopaedic effects of shoe gear using a medical team composed of orthopaedists, it required IRB approval. Accordingly, only recruits that volunteered and signed informed consent participated, and they had the right to withdraw from the study at any time. Because so many soldiers dropped out quickly without allowing their shoes to “break in”, the study methodology could not be completed. Testing boots, apparel or similar hazardless equipment is usually performed by the TLB without involving medical staff and does not use a randomized controlled trial design. Therefore IRB approval is not required. When soldiers are aware of testing, it introduces biases (particularly a type of “position bias” where the new is assumed to be better). While using a study design that does not require IRB approval and informed consent has many drawbacks, it has a major advantage in terms of study completion. If the recruits in this MDIP study were issued boots without a choice as to whether to use them or not, it is likely that they would have completed their designated training with them. The boot could have then been evaluated by a questionnaire administrated by non-medical staff at the end of the training period and fuller boot evaluation achieved than that of our study. It is clear that designing the current study as a “medical intervention” necessitating medical regulations was not the optimal choice for initial evaluation of the new military boot [8].

It can be said that the soldiers in this study voted with their feet against the NDIB and for the HWIB. When first issued the NDIB boots, recruits had already worn the hot weather infantry foot for almost 2 weeks. Although this biased their experience with the NDIB boot, it also gave them a standard for comparison. They reported almost immediately that the NDIB boot was much less comfortable. 81 % experienced problems with the NDIB within the first 9 days. The feet of all recruits were examined and evidence of traumatic skin lesions were found in many of the NDIB wearers. This may be a function of its hard leather upper and/or the boot last. Male shoes are generally built around a last which is D width. The distribution of the shoe widths as measured by the Brannock device indicates that many of the Israeli recruits in this study had narrower feet than the standard D shoe lasts. Their shoe widths generally tended to be narrower than their counterparts in the Canadian Army [9]. The data indicates that a standard D last shoe is too wide for more than 50 % of the recruits in this study. Inappropriate shoe fit can lead discomfort and to overuse injuries [10].

## Conclusions

No advantages to the NDIB were identified in the present study. Greater durability might be expected, but we were not able to assess this variable due to the high dropout and early termination of the study. The durability of HWIB when compared to the IDF boot it replaced is lower, probably related to the bilayer rubber sole of the previous boot and various differences to the upper that make the HWIB more like a sports shoe than a safety boot, the traditional concept of military boots. These differences are clearly preferred by the recruits and related to less “breaking in” injuries in basic training. The price of this improvement is less durability. It is also possible that if the study had been done in cold wet weather, the recruit response to the NDIB boot might have been more favorable. Although the HWIB were well liked by the IDF infantry recruits in this study it remains to be determined if the boots are appropriate for IDF reserve army service.

## Abbreviations

HWIB: hot weather infantry boots
NDIB: newly designed Israeli infantry boots
IDF: Israel Defense Forces
TLB: Technological and Logistics Branch
NCO: base non-commissioned officer
RCT: randomized controlled trial
IRB: institutional review board
